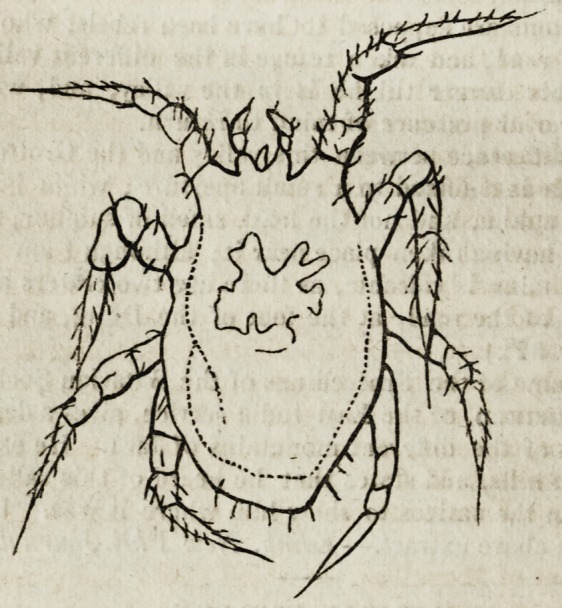# Collectanea

**Published:** 1832-12

**Authors:** 


### COLLECTANEA.

**Floriferis ut apes in saltibus omnia libant,**
**Omnia nos, itidem, depascimur aurea dicta.**

### PATHOLOGY.

*Tuberculous Phthisis cured: Tubercle expectorated.* An English gentleman, aged thirty-six, detained in Paris as prisoner of war, in September 1813 had an attack of hæmoptysis, followed by a cough, at first dry, but in the course of a few weeks accompanied by purulent sputa. To these symptoms were added a well-marked hectic, considerable dyspnœa, copious night sweats, emaciation, and great debility. The chest sounded well everywhere, except under the right clavicle, and in the axilla of the same side. The hæmoptysis returned in a slight degree now and then; and, in December, he had diarrhœa, which was with difficulty checked by astringents. In the beginning of January, he was so much reduced that M. Hallé and Bayle agreed with me in opinion that his death might be daily looked for. On the 15th of January, during a severe fit of coughing, and after bringing up some blood, he expectorated a solid mass, of the size of a filbert, which, on examination, I found to be evidently a tubercle in the second stage, surrounded apparently by a portion of the pulmonary tissue, such as has already been described as impregnated with grey tubercular matter, in the first stage often met with around these bodies when large.

This patient remained in the same degree of extreme emaciation and debility all January, being expected to die daily; but, in the beginning of February, the perspirations and diarrhœa ceased spontaneously; the expectoration sensibly diminished, and the pulse, which had been constantly as high as 120, fell to 90. In a few days the appetite returned, the patient began to move about in his room; his emaciation became less; and, against the end of the month, his convalescence was evident. In the beginning of April he was perfectly recovered, and his health has continued good ever since, without even the least cough, and without his being at all particularly guarded in his climate or regimen.

In 1818, this patient again consulted me for a different complaint, and I took the opportunity of examining his chest by means of the stethoscope. The only thing I could detect was the comparative indistinctness of respiration in the superior portion of the right lung, as low as the third rib. This part, however, sounded as well on percussion as the opposite side, and there was no pectoriloquism.

From these circumstances I am of opinion, that the excavation which contained the expectorated tubercle must have been replaced by a cellular or fibro-cartilaginous cicatrix; and as the total absence of cough, dyspnœa, and expectoration, for so long a period, forbids the supposition of the existence of others in the lungs, I think we have a right to consider this patient as perfectly cured. In 1824, this gentleman was examined at Rome by Dr. CLARK, an English physician, who practises there with great distinction, and who recognised him as the subject of the case just detailed. I saw him also the same year, and found him precisely in the same state as in 1818.—FORBES' *Translation of* LAENNEC *on Diseases of the Chest.*

---

*Death from an Irritating Injection into the Abdomen.* M. DUPUY had for many years treated a female, aged twenty-seven years, for encysted dropsy of the abdomen: she had been tapped five times. He wished to try the radical cure, by inflaming moderately the inner surface of the cyst. For this purpose, after having made a puncture, he adapted a gum elastic tube to the canula of the trocar, and, by means of this apparatus, he passed into the cyst the vapour of heated wine. Inflammation set in certainly, but with rather more violence than M. Dupuy had calculated on: it extended beyond the membrane of the sac, and after thirteen days the girl died of general peritonitis. The autopsy was not allowed, but, on puncturing the abdomen, exit was given to a large quantity of yellow fetid matter.—*Journal de la Societé Med. de Bourdeaux; and Dublin Journal of Med. and Chem. Sciences.*

---

*Contributions to Pathological Anatomy.* By RICHARD TOWSEND, M.D., M.R.I.A., Fellow and Censor of the King and Queen's College of Physicians, Senior Medical Inspector of the House of Industry, Lecturer on Pathological Anatomy, &c.

CASE I. *Sudden Death. Spontaneous Rupture of the Heart.* The body of a very old woman was brought into the dead-room of the Whitworth Hospital, for anatomical examination, on the 30th of August, 1830. The external appearance of the body did not in any respect indicate previous disease: on removing the sternum, the pericardium appeared unusually prominent, and of a bluish white colour. When opened, it was found to contain more than half a pint of dark clotted blood, which completely enveloped the surface of the heart. When this coagulum was removed, the heart appeared of its natural size, but was enormously laden with fat, especially at its basis and over the right ventricle. On the anterior surface of the left ventricle, near its septum, and at the distance of about an inch from the apex, a longitudinal fissure, half an inch in length, was discovered, the edges of which were jagged, and had evidently been separated by tearing; there was a slight degree of ecchymosis under the serous membrane, in the immediate neighbourhood of the wound. On laying open the left ventricle, it was found that the fissure seen on the external surface extended, through the fat and muscular substance, into the interior of the left ventricle. This cavity was quite empty, all its blood having previously escaped through the wound. The length of the fissure on the internal surface of the ventricle was somewhat greater than on the external surface, but corresponded with it exactly in other respects, being a mere cleft or clink just wide enough to admit the handle of a scalpel. The lining membrane in the neighbourhood of the rupture was soft and friable, and the columnæ carneæ, for about the circumference of a shilling, were of a dull white colour, and so soft as to break down under the scalpel. The left ventricle was of its natural dimensions, and its parietes of their ordinary thickness, but the muscular fibres were pale, soft, and flabby. The other cavities of the heart presented their usual appearance, except that the muscular walls of the right ventricle were as thin as paper, and coated with a layer of fat nearly half an inch deep. The valves were all remarkably healthy for a subject so far advanced in life. The coronary arteries exhibited several patches of atheromatous deposit, sufficient in many points to diminish their caliber considerably. The arch of the aorta was dilated and atheromatous. The other viscera were all healthy, and the muscles of the trunk and other extremities appeared even more firm and florid than is usually observed in persons of her great age.

Upon inquiring into the previous history of this individual, it was ascertained that she was ninety years of age, and had been a servant in the house of industry for many years; her usual occupation was that of scouring the floors; she always enjoyed excellent health, with the exception of an occasional slight cough. In her 88th year she fractured her thigh near the trochanter, but completely recovered from the effects of that accident in the usual time.

On the morning of her death, she went to the chapel in as good health and spirits as usual, and, while in the act of saying her prayers, she suddenly dropped down dead without a struggle or a moan.

Case II. *Sudden Death. Spontaneous Rupture of the Heart.* Another case, of a similar nature to the preceding, occurred in the same establishment on the 5th of March, 1832. The subject of it was a large athletic man, ætat. eighty-four, who had served twenty-six years in the navy, and after a long life of hardships and vicissitudes, was admitted four years ago into the pauper wards of the House of Industry. During the time of his residence in this establishment, his health was uniformly excellent, with the exception of a slight sensation of uneasiness which he occasionally felt in the left side, in the region of the heart. The night before his death, he complained of a return of this pain, and of difficulty of breathing. Next morning these symptoms had totally disappeared, and he got up at his usual early hour, in as good health and spirits as he had enjoyed for many months previously. He went to chapel, walked afterwards in the yard for about two hours, returned to his ward, and while in the act of cutting tobacco at the end of the table, and in conversation with one of his comrades, he suddenly fell down, and expired instantaneously. His family are represented to be remarkably long-lived; there is a sister of his at present in the House of Industry, in her eighty-sixth year.

On opening the body, the only morbid appearances were found in the thorax. The parenchymatous structure of the lungs was rarified and emphysematous, as is usually the case in persons of his time of life. The pericardium was greatly distended, and occupied the principal space of the lower part of the left side of the thorax. On slitting it open, a large quantity of black blood, partly coagulated and partly fluid, was found occupying its interior. The heart was rather larger than the fist of the patient, but its increase of size was chiefly attributable to a large quantity of fat which was developed on its surface, principally about the origin of the great blood-vessels and along the septum ventriculorum, in both which situations it measured near half an inch in thickness. The muscular substance of the heart was generally pale, exsanguineous, and flabby; in the right ventricle the walls were as thin as paper; in the left, they retained their usual thickness. The coronary arteries were ossified, and the ossification extended even to the minute ramifications which went to the apex of the heart. On the anterior surface of the left ventricle, about an inch from the septum, and the same distance from the apex, a rupture of the parietes was discovered, half an inch long, and several lines wide: this opening was nearly closed by one of the columnæ carneæ, which was torn across and projected through the external orifice. The muscular fibres in the vicinity of the rupture were in a state of perfect ramollissement, but neither on the external or internal surface was there any unequivocal appearance of ulceration. There were two patches of ecchymosis as large as half-crowns, under the serous membrane on the surface of the left ventricle. No other morbid appearances were observed. Not the least remarkable circumstance in the history of these two cases is the sudden occurrence of the fatal accident in the midst of apparently sound health, not preceded by any premonitory symptoms, or brought on by any moral emotion, or physical exertion. But, though there were no evident symptoms of organic disease manifested during life, there was abundant evidence, on dissection, that the structure of the heart was materially altered from its natural condition. The coronary arteries were extensively diseased in both cases, and the supply of arterial blood which they transmitted was, consequently, inadequate for the due nutrition of the muscular fibres of the organ, which were accordingly soft, pale, and flabby; and, as usually happens in such cases, an inordinate quantity of fat was deposited on the surface of the heart, as if to compensate for the atrophy of its muscular substance.

In the greater number of cases where rupture of the heart has been observed to occur, it has been immediately preceded by some moral emotion, or physical exertion, or concussion, as its immediate cause, to which the accident is generally attributed. Nichols, who gives a very clear and succinct account of the rupture of the heart which caused the death of George the Second, attributes the accident to the efforts made while straining at stool.—*Phil. Trans.*, vol. lii. part I.

A similar accident terminated, not long since, the existence of a late eminent barrister of this city. Bertin mentions a case of rupture of the right auricle, caused by falling out of a window; and Haller and Morgagni have known the heart ruptured during violent paroxysms of pain, and the convulsions of epilepsy. These causes, however, can scarcely be conceived sufficient to produce such a formidable effect, unless where the organ was predisposed to rupture by some preceding lesion; and, where such predisposing cause is strongly marked, the ordinary force of the heart's contractions may be sufficient to determine its rupture, as in the present instances.

From the reports which have hitherto been published of this fatal accident, it appears that the true cause of the rupture is most commonly attributable to the parietes having been previously eaten away, to a certain depth, by ulceration. The ulcerative process may commence either at the external or internal surface of the heart, or may attack both surfaces at the same time, and continue to burrow until both ulcers meet, and so complete the perforation. This species of rupture may be considered analogous to the perforation of the stomach and intestines, from the successive ulceration of their coats.

Andral mentions, in his Treatise on Pathological Anatomy, that the French pathologists generally enumerate the excessive deposition of fat on the surface of the heart among the causes which predispose to its rupture; but this opinion, he conceives, should receive further confirmation before it is finally adopted, as the only case in point to establish the fact is that related by Dr. Gratiloup, of Bourdeaux, (in the Archives Generales for 1822,) of a curate of that town, who died suddenly in his bed of rupture of the right auricle, the heart being at that time prodigiously laden with fat. The case, which I have related would seem to support the doctrine that the excessive deposition of fat on the surface of the heart, especially when accompanied, as it usually is, by atrophy and softening of the muscular fibre, may render it susceptible of being easily torn and ruptured. But, even admitting this supposition, it still remains to be explained why it is not in the auricles, or in the right ventricle, where the muscular parietes are thinnest, and the fat most abundant, that the rupture generally occurs; but about the middle of the left ventricle, where the fat seldom accumulates in any great quantity, and the muscular coat is particularly thick and strong. This circumstance would seem to prove that the rupture of the parietes in these cases should not be regarded as a mere mechanical effect produced by their overdistention, but rather as an active process in some degree analogous to the rupture of the uterus, from excessive contraction of its fibres.

Perforation of the heart, from whatever cause it proceeds, is generally followed by immediate death. Bertin states, that of ten cases of this accident, eight died instantaneously, and the other two after a few hours. The extent of hemorrhage into the pericardium cannot account for these fatal consequences, as the quantity of blood effused is often very trifling. The great and sudden shock which the nervous system sustains is more probably the immediate cause of death. In some rare cases, death does not take place for several days after the accident. It is stated that, in these cases, the perforation has been found plugged up with a coagulum of fibrine. (*Andral.*)—*Ibid.*

---

*Death from Asphyxia, caused by large Tuberculous Masses developed in the Parietes of the Left Auricle, compressing the Trunks of the Pulmonary Veins, so as to reduce their Diameter to that of a Crowquill, thereby preventing the Return of the Blood from the Lungs.* From "Contributions to Pathological Anatomy," by R. Townsend, M.D. M.R.I.A. &c. (*Dublin Journal of Medical and Chemical Sciences.*)

John Larkin, a poulterer, æt. sixty-two, states, that he has occasionally suffered from cough and shortness of breathing for several years past, but that he never felt seriously ill until about twelve months since, when, after putting on damp clothes, he was seized with cough, dyspnœa, palpitations, and profuse hæmoptysis: for these symptoms he was bled copiously; the hæmoptysis ceased after the second week, and has not since returned; the cough likewise abated considerably, but the palpitations and dyspnœa continued to recur at irregular intervals throughout the remainder of the winter and spring; during the summer he was so much better as to resume his ordinary occupations, but since the commencement of the present winter (1829,) his breathing has become habitually short, and at times he has been so oppressed, for hours together, as to make him suppose each succeeding gasp must be his last; states, that these violent paroxysms are brought on by changes of weather, flatulence, or mental vexations; but never had chills, night-sweats, nor diarrhœa.

The following is a summary of the principal symptoms which he presented during his stay in the Whitworth Hospital, to which he was admitted on the 18th of December, 1829. He was excessively emaciated, and was constantly annoyed with a short, dry, harassing cough, his breathing was extremely variable, and, as he himself remarked, materially affected by the state of the weather: during the two or three frosty days that occurred in the first week of January, he was so well as to walk down stairs and take several turns in the garden; but during the damp weather which succeeded, his breathing was frightfully oppressed in paroxysms, resembling the most violent attacks of spasmodic asthma. His pulse was never less than one hundred in the minute, weak, compressible, and regular. The heart's action, as heard by the stethoscope, was extremely feeble, and could with difficulty be heard fluttering faintly, and apparently at a great distance behind the sternum; in the epigastrium its action was rather more perceptible, but its sound and impulse were so excessively feeble and indistinct, that it was impossible to analyze them. The sound on percussion was remarkably dull all over the thorax. During the last fortnight of his existence, no respiration could be heard in any part of the left lung; in the right it was puerile for about two fingers' breadth under the clavicle, lower down it was extremely feeble, and almost completely marked by a subcrepitating râle. At this period he was obliged to sit bolstered up with pillows, and had regularly a frightful paroxysm of suffocation at midnight. His strength now failed him completely; his face assumed a livid cadaverous aspect; his ideas wandered, and became incoherent; and he died asphyxiated about five weeks after his admission into hospital.

Of all the remedies which were employed, bloodletting alone seemed to afford him even temporary relief, but the smallest abstraction of blood was, latterly, followed by such extreme prostration of strength, that its employment was renounced altogether. Antispasmodics and counter-irritants seemed to produce no effect whatever on his symptoms.

On dissection made twelve hours after death, a considerable quantity of serous fluid was found effused on the surface of the brain. On opening the thorax, the lungs appeared to fill the cavity completely, the superficial cells on their anterior surface were considerably dilated, laterally and posteriorly; the lungs at each side were firmly attached to the costal pleura; the medium of attachment was of the colour and consistence of cartilage. On removing the lungs from the chest, they appeared externally of a dark red colour, conveyed a distinct sense of fluctuation to the finger, and felt remarkably heavy. When the left lung was cut into, an immediate gush of blood followed, as if the incision had been made into an aneurismal sac. The quantity of blood which escaped could not be less than three pints and a half; after it had been removed, the pulmonary veins from which it flowed were seen traversing the parenchyma of the lung, dilated to at least four times their natural size; those veins which are naturally no bigger than crowquills, being as large as the fingers of a glove. On tracing the dilated veins towards the root of the lung, the dilatation was found to extend uniformly from the smallest branches to the main trunks, which formed two large sinuses outside the left auricle. The right lung presented a similar appearance, but in a minor degree. On examining the heart, the dilatation and congestion of the pulmonary veins were found to arise from the compression which they suffered at their entrance into the left auricle, the parietes of which appeared converted into one solid unyielding mass of tuberculous matter, nearly one inch in thickness: this morbid production was developed between the outer and inner membranes of the auricle, and, by the pressure which it made on the pulmonary veins, diminished their caliber so much, that a probe could with difficulty be passed through them into the auricle. The consequence of this constriction of the veins at their orifice was, that the blood, which was thus obstructed in its return to the heart, accumulated in the trunks and minor branches of the veins in such quantities as to produce the enormous dilatation of those vessels which I have endeavoured to describe. The right auricle and ventricle were considerably dilated; the heart in other respects appeared healthy. The bronchial glands were much enlarged, and filled with tuberculous matter. The lungs contained only a few miliary tubercles. The abdominal viscera did not present any unusual appearance.

I have thought this case deserving of being recorded, as it serves to illustrate a morbid condition of the heart and lungs of extremely rare occurrence, and likewise as exemplifying a pathological fact, which I have had repeated opportunities of observing, namely, that a permanent organic lesion may give rise to symptoms of a remittent, or even of an intermittent character. In this instance the mechanical obstruction to the pulmonary circulation, and the consequent congestion of the pulmonary tissue, must have opposed a constant obstacle to the due aëration of the blood, and yet the symptoms of distress were only felt occasionally, or at least were greatly aggravated at intervals. In like manner, I have known aneurism of the abdominal aorta distend the nervous filaments of the solar plexus to such a degree as to lacerate several of them, and yet the pain experienced in this case occasionally intermitted for whole weeks together.

I have searched in vain in modern authors for the description of a state of the heart and lungs, such as I have recorded; but in Sprengel's erudite History of Medicine, I find mention made of a case almost precisely similar, which was reported by Maurocordatus, a Turkish physician, in the year 1664, and is thus described by Sprengel (Histoire de la Médecine, traduite par A. J. L. Jourdan, tom. iv. p. 129.) " Aux vingt-six raisons qui Maurocordatus allègue en faveur de la circulation pulmonaire, il ajoute encore une observation faite par lui-même sur le cadavre d'un de ses maîtres. Cet homme était mort d'un asthme suffocatoire, on trouva les poumons singulièrement distendus, l'oreillette pulmonaire cartilagineuse, le ventricule gauche vide, mais les veines pulmonaires gorgées de sang; il en conclut que ces dernières raminent le fluide du poumon." From this description it appears, that not only was the organic lesion similar, but that the symptoms which it produced were likewise those of spasmodic asthma.

---

### MIDWIFERY.

*Extraordinary Birth.* On the 30th of December, 1831, the wife of a man named Dernian Ploson, living in the village of Dropin, in Bessarabia, was delivered of *six daughters*, (the fruits of one pregnancy,) all living, and only a little smaller than the usual size of children at birth, with the exception of the last, which was much the least. The mother is not quite twenty years of age, and of a strong constitution. The whole six children lived long enough to be baptised, but died in the evening of the day of their birth. The mother suffered from a severe indisposition subsequent to her confinement, but is now quite well.—*Gaz. Médicale.*

---

### NATURAL HISTORY.

*Visit to the Valley of Death, in the Island of Java.* By A. Loudon, Esq., in a Letter to Professor Jameson. (*Extract from " Journal of a Tour through the Islands of Java and Madara, last year."*)

"Balor, 3d July, 1830. This evening, while walking round the village with the Pattet, (native chief,) he told me that there was a valley, only three miles from Balor, that no persons could approach without forfeiting their lives, and that the skeletons of human beings, and of all sorts of beasts and birds, covered the bottom of the valley. I mentioned this to the commandant, M. Van Spreewenberg, and proposed our going to see it: M. Daendels, the assistant resident, agreed to go with us. At this time I did not credit all that the Javanese chief told me. I knew that there was a lake close to this that it was dangerous to approach too near, but I had never heard of the Valley of Death.

Balor, 4th July. Early this morning we made an excursion to the extraordinary valley called by the natives " Guwo Lipas," or Poisoned Valley: it is three miles from Balor, on the road to the Djiang. M. Daendels had ordered a footpath to be made from the main road to the valley. We took with us two dogs and some fowls, to try experiments in this poisonous hollow. On arriving at the foot of the mountain, we dismounted, and scrambled up the side about a quarter of a mile, holding on by branches of trees, and we were a good deal fatigued before we got up; the path being very steep and slippery, from the fall of rain during the night. When within a few yards of the valley, we experienced a strong, nauseous, suffocating smell; but on coming close to the edge, this disagreeable smell left us. We were now all lost in astonishment at the awful scene before us. The valley appeared to be about half a mile in circumference, oval, and the depth from thirty to thirty-five feet; the bottom quite flat; no vegetation; some very large (in appearance) river stones; and the whole covered with the skeletons of human beings, tigers, pigs, deer, peacocks, and all sorts of birds. We could not perceive any vapour or any opening in the ground, which last appeared to be of a hard, sandy substance. The sides of the valley, from the top to the bottom, are covered with trees, shrubs, &c. It was now proposed by one of the party to enter the valley; but at the spot where we were, this was difficult, at least for me, as one false step would have brought us to eternity, and no assistance could be given. We lighted our cigars, and, with the assistance of a bamboo, we went down to within eighteen feet of the bottom. Here we did not experience any difficulty in breathing, but an offensive nauseous smell annoyed us. We now fastened a dog to the end of a bamboo, eighteen feet long, and sent him in; we had our watches in our hands, and in fourteen seconds he fell on his back, did not move his limbs or look round, but continued to breathe for eighteen minutes. We then sent in another, or rather he got loose from the bamboo, but walked in to where the other dog was lying: he then stood quite still, and in ten seconds he fell on his face, and never moved his limbs afterwards; he continued to breathe for seven minutes. We now tried a fowl, which died in one minute and a half. We threw in another, which died before touching the ground.

During these experiments we experienced a heavy shower of rain, but we were so interested by the awful scene before us, that we did not care for getting wet.

On the opposite side, near a large stone, was the skeleton of a human being, who must have perished on his back, with his right arm under his head: from being exposed to the weather, the bones were bleached as white as ivory. I was anxious to procure this skeleton, but any attempt to get at it would have been madness.

After remaining two hours in the Valley of Death, we returned, but found some difficulty in getting out. From the heavy shower, the sides of the valley were very slippery, and, had it not been for two Javanese behind us, we might have found it no easy matter to escape from this pestilential spot. On reaching our rendezvous, we had some brandy and water, and left this most extraordinary valley; came down the slippery footpath, sometimes on our hams and hands, to the main road; mounted our horses, and returned to Balor, quite pleased with our trip.

The human skeletons are supposed to have been rebels; who had been pursued from the main road, and taken refuge in the different valleys; as a wanderer cannot know his danger till he is in the valley, and, when once there, one has not the power or presence of mind to return.

There is a great difference between this valley and the Grotto del Cane near Naples, where the air is confined to a small aperture; while here the circumference is fully half a mile, and not the least smell of sulphur, nor any appearance of an eruption having taken place near it; although I am aware that the whole chain of mountains is volcanic, as there are two craters at no great distance from the side of the road, at the foot of the Djing, and they constantly emit smoke. (Fahr. 52°.)

In the eighth volume of the Proceedings of the Batavian Society of Arts and Sciences, Dr. Horsefield, of the East-India service, gives a description of the mineral constitution of the different mountains of Java. He examined several parts of the chain of hills, and states that he heard of this valley, but that he could not prevail on the natives to shew him where it was. I have sent the Doctor a copy of the above extract.—*Edinb. New Phil. Journal.*

---

### MEDICAL ZOOLOGY.

*A new Acarus; described by* Bory de St. Vincent. A female had been in a bad state of health for fifteen years, which no remedy had in any way relieved. She was ultimately attacked by dropsy, for which she applied to another physician, who apparently restored her, and she fancied herself free from all her former complaints: but, as she seemingly improved in her general health, she experienced an itching all over her body, so that she was constantly obliged to scratch the prurient spots, till she produced fissures in the skin, out of which exceedingly minute, brown-coloured animalculæ issued, which ran by thousands over her whole skin, but seemed particularly fond of settling in any cotton garment she had near. She consequently wrapped herself entirely up in muslin; but, according to the degree of heat of her body, she was obliged to change it from three to six times a day, so immense was the number of these creatures.

She died a fortnight after this attack, without being emaciated; but her skin had a glossy appearance.

The animalculæ which were brought to me lived about forty-eight hours; most of them were barely visible, and the largest were only half the size of a tobacco-seed.

The margin of the body and the feet were covered with stiff articulated bristles; the mouth has no mandibulæ, and is more like a proboscis; it projects a little, and is generally hidden between the two blunt-pointed pulpi, which move quite horizontally, and which, together with the mouth, appear to be a process of the body, between the two eminences from which the two front legs arise. Otherwise, there is no division between the head and the body, and none at all between the thorax and abdomen. Eight slightly, but yet distinctly articulated legs, with the last joint narrower but longer, having no unguis, but at the end a longer and stiffer bristle; which, however, is wanting in some. The front pair of legs are the longest, and are placed more underneath the body than the other three pair, which are more at the side: they serve not only for walking, but are often bent slightly forwards, as in the arachnoidæ with clavated palpi. In the middle of the body is a reddish-coloured spot.

The insect appears so be formed by a congeries of very minute globules, connected by an outer integument of skin, which protrude on pressure. There is no web perceptible. It belongs to the Acaridæ, and has some resemblance to the Smaris, which, however, has eyes and short inarticulated palpi; otherwise it is like the Acaris scabiei, but it has certainly no mandibulæ. Annexed is a much magnified representation.—*Annal. des Sciences.*

## Figures and Tables

**Figure f1:**